# Comprehensive Genomic Profiling of Relapsed Interdigitating Dendritic Cell Sarcoma Reveals Novel Molecular Insights in a Patient Achieving Durable Remission After Autologous Stem Cell Transplantation

**DOI:** 10.3390/ijms27219474

**Published:** 2026-10-23

**Authors:** Costantino Riemma, Chiara Cavatorta, Chiara Salvesi, Jessica Garbetta, Davide Angeli, Eugenio Fonzi, Federico Lo Schiavo, Chiara Molinari, Giorgia Simonetti, Francesco Malaspina, Jessica Rosa, Gerardo Musuraca, Anna Ferrari

**Affiliations:** 1 Hematology Unit, IRCCS Istituto Scientifico Romagnolo per lo Studio e la Cura dei Tumori (IRST) “Dino Amadori”, 47014 Meldola, FC, Italy; 2 Pathology Unit, “Maurizio Bufalini” Hospital, 47521 Cesena, FC, Italy; 3 Biosciences Laboratory, IRCCS Istituto Romagnolo per lo Studio dei Tumori (IRST) “Dino Amadori”, 47014 Meldola, FC, Italy; 4 Unit of Biostatistics and Clinical Trials, IRCCS Istituto Romagnolo per lo Studio dei Tumori (IRST) “Dino Amadori”, 47014 Meldola, FC, Italy

**Keywords:** interdigitating dendritic cell sarcoma (IDCS), autologous stem cell transplantation (ASCT), next-generation sequencing (NGS), *BRAF* L597R, *SMARCA4*

## Abstract

Interdigitating dendritic cell sarcoma (IDCS) is an extremely rare hematologic malignancy with no standardised therapeutic approach, particularly in the relapsed setting. We report a case of a 69-year-old woman with multifocal relapsed IDCS, *KRAS/BRAF/SMARCA4* mutated, successfully treated with CHOP chemotherapy followed by consolidation with high-dose chemotherapy and autologous stem cell transplantation (ASCT), achieving a durable complete remission. This case supports the potential role of intensive chemotherapy and ASCT in selected patients with aggressive or relapsed disease.

## 1. Introduction

Interdigitating dendritic cell sarcoma (IDCS) is an ultra-rare and aggressive hematologic malignancy arising from antigen-presenting interdigitating dendritic cells. Under physiological conditions, interdigitating dendritic cells have an important role in the initial part of the immune response, as they present antigens to T cells. They are distributed within the paracortex and deep cortex of lymph nodes, in periarteriolar lymphoid sheets of the spleen and also in mucosa-associated lymphoid tissue. They originate from hematopoietic precursors, including Langerhans cells, myeloid and lymphoid precursors [1].

According to the current World Health Organisation and International Consensus Classification, IDCS belongs to the family of histiocytic and dendritic cell neoplasms and, together with histiocytic sarcoma and Langerhans cell sarcoma, represents one of the malignant histiocytic neoplasms (MHNs). These extremely low-incidence neoplasms are characterised by an aggressive clinical course, with high relapse rates, poor overall survival, and extremely low incidence [2,3]. In some cases, malignant histiocytic neoplasms occur secondary to or in association with another hematopoietic malignancy, such as a trans-differentiated, treatment-resistant clone [4]. Since only a limited number of cases have been reported worldwide, the natural history, prognostic factors and optimal therapeutic management of IDCS remain poorly defined [1].

IDCS predominantly involves lymph nodes but may also arise in extranodal sites, including the head and neck, gastrointestinal tract, skin, liver and spleen [1,5]. Bone marrow involvement has been reported in a minority of cases, manifesting with cytopenias and, rarely, hemophagocytosis [4]. Clinical presentation ranges from localised disease, often amenable to complete surgical excision, to disseminated or relapsed disease with rapid progression and poor survival [1,5]. Several clinicopathologic studies have suggested that tumour size, intra-abdominal involvement, disseminated disease and relapse after initial therapy are associated with inferior outcomes [1,5]. However, because available evidence derives almost exclusively from isolated case reports and small retrospective series, reliable prognostic models are still lacking.

The diagnosis of IDCS remains particularly challenging because no disease-specific immunophenotypic marker has been identified. Histologically, the tumour consists of spindle to epithelioid cells arranged in fascicles or whorls within a background of inflammatory cells. Immunohistochemistry plays a pivotal role in establishing the diagnosis, with tumour cells typically expressing S100, vimentin, and HLA-DR, with variable expression of CD68 and CD163, while lacking expression of follicular dendritic cell markers (CD21, CD23 and CD35), Langerhans cell markers (CD1a and langerin) and lineage-specific lymphoid markers [1,5]. This broad morphologic and immunophenotypic overlap with other hematologic, mesenchymal and melanocytic malignancies frequently delays diagnosis and contributes to the rarity of well-characterised cases.

**Figure 1:**
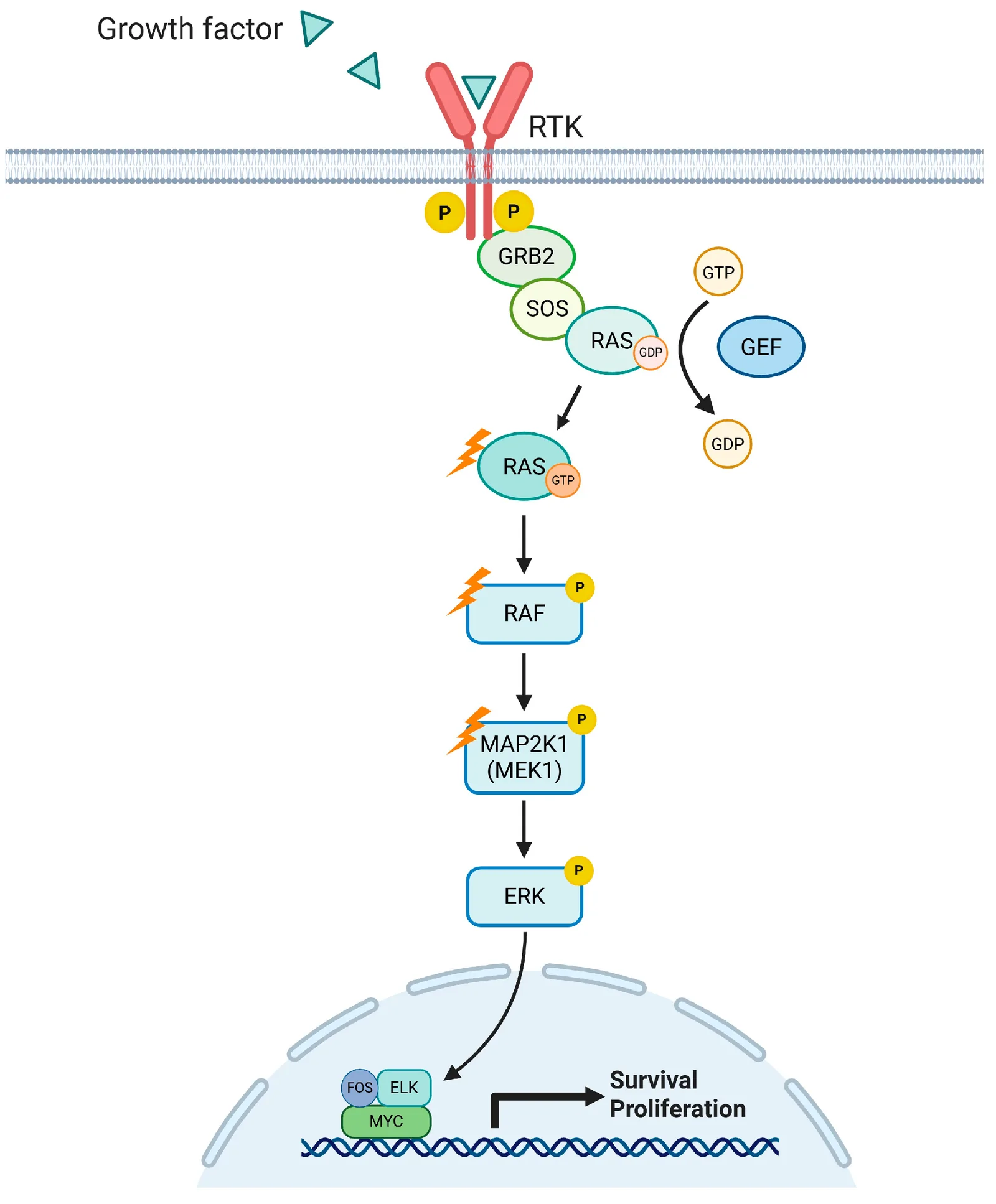
The MAPK signalling cascade involved in the pathogenesis of interdigitating dendritic cell sarcoma (IDCS) is shown. Proteins that may be affected by mutations are highlighted with lightning bolts. Mutations in *RAS* may contribute to disease pathogenesis and progression. One of the most frequent mutations affecting *BRAF* is the activating V600E mutation, which is commonly observed in histiocytoses. In cases in which *BRAF* is wild-type, mutations or overexpression of *MAP2K1* have been frequently reported, including in hairy cell leukaemia, melanoma, and carcinomas [8,11].

Given the rarity of IDCS and other malignant histiocytic neoplasms, treatment recommendations remain largely based on retrospective case series in the absence of prospective clinical trials. A recent expert consensus has been developed by the Histiocyte Society’s Working Group on Malignant Histiocytic Neoplasm [1,4]. Treatment is primarily guided by disease extension. Complete surgical resection is generally recommended for localised diseases. Adjuvant radiation therapy may be considered as an option if there are positive margins or concerns for incomplete resection [1,4,5,6]. For multifocal/disseminated disease, systemic chemotherapy used for aggressive lymphomas, most commonly cyclophosphamide, doxorubicin, vincristine, and prednisone (CHOP) or CHOP-like-based regimens, ABVD, ICE, ESHAP, DHA, with or without radiotherapy, is commonly employed [1,4,5,6]. Emerging evidence of PD-L1/PD-L2 expression in MHNs raises the potential therapeutic role for immune checkpoint inhibitors in these neoplasms [4]. Nevertheless, therapeutic responses are highly heterogeneous and frequently short-lived, particularly in patients experiencing relapse. Consequently, no standard of care has been established for recurrent IDCS, and the role of hematopoietic stem cell transplantation remains largely undefined, being supported only by isolated reports describing durable remissions in selected patients [1,6]. Recent advances in the molecular characterisation of histiocytic and dendritic cell neoplasms have improved the understanding of their pathogenesis while also revealing potentially actionable molecular vulnerabilities. Although the genomic landscape of IDCS remains incompletely defined, recurrent alterations converging on the MAPK signalling pathway appear to represent a common biological hallmark. Activating *BRAF* mutations, particularly the V600E variant, have been described in a subset of IDCS cases, whereas *MAP2K1* mutations or overexpression represent the most frequent alterations among *BRAF* wild-type tumours [7,8,9]. Additional activating mutations involving other components of the MAPK cascade, including *KRAS*, *NRAS* and *MAP3K1*, further support the central role of this pathway in disease pathogenesis and may contribute to the lineage plasticity underlying trans-differentiation from pre-existing lymphoid malignancies reported in a subset of cases (Figure 1) [8,9]. Beyond MAPK signalling, isolated reports have identified alterations affecting genes involved in epigenetic regulation and receptor tyrosine kinase signalling, including *KMT2D*, *SETD2*, *SF3B1*, *MET* and *ERBB3*, as well as amplifications of *PDGFRA* and *KIT*, suggesting that the molecular heterogeneity of IDCS extends beyond a single oncogenic pathway [8]. Nevertheless, genomic data specifically addressing IDCS remain remarkably limited, as only a handful of cases have been investigated using next-generation sequencing (NGS) or other high-throughput approaches [10]. Consequently, the prevalence, biological significance and therapeutic implications of many of these alterations remain largely unknown, highlighting the need for comprehensive molecular profiling to improve disease classification and identify novel precision medicine strategies.

Here, we report the clinical course and comprehensive molecular characterisation of a patient with relapsed IDCS treated with intensive chemotherapy followed by consolidation with autologous stem cell transplantation (ASCT), providing additional insights into the management and molecular landscape of this ultra-rare malignancy. 

## 2. Case Description

A 69-year-old Caucasian woman was referred to our institution in July 2021 with relapsed IDCS, occurring one year after complete surgical excision of a primary lesion involving the left tongue hemibase alongside a left cervical lymph node, performed at another centre.

**Figure 2:**
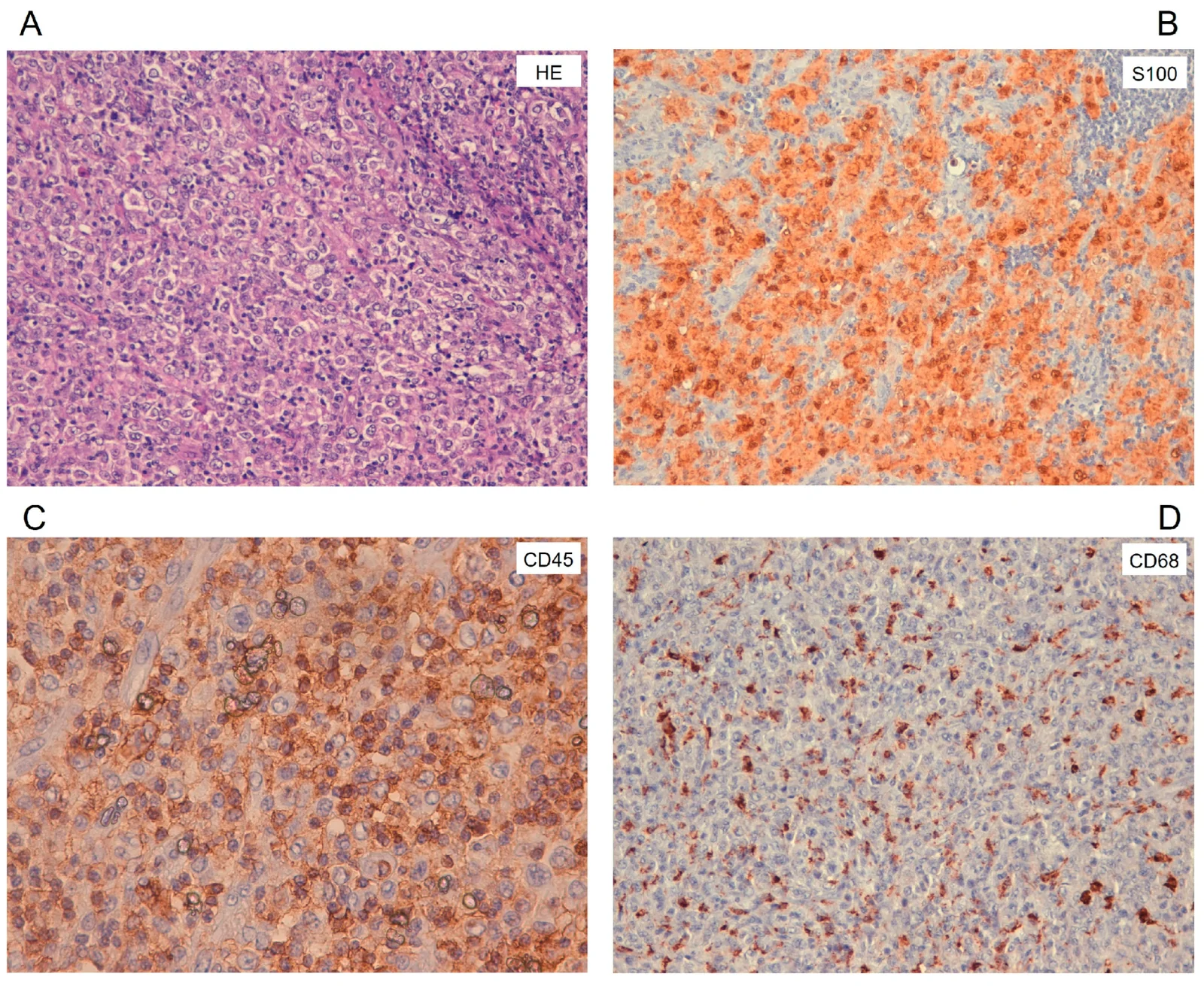
Cervical lymph node sample at disease diagnosis. (**A**) Epithelioid morphology on haematoxylin and eosin staining (20×); immunohistochemical analyses of (**B**) S100 (20×); (**C**) CD45 (40×, weak signal); and (**D**) CD68 (20×, dot-like pattern) markers.

Her past medical history was unremarkable. In May 2020, the patient initially presented with an ulcerated mass at the left tongue base and ipsilateral cervical lymphadenopathy. Biopsy revealed a CD45-positive malignant neoplasm of lymphoid origin, and subsequent lymph node excision established, following expert review at the regional reference centre, a diagnosis of interdigitating dendritic cell sarcoma. The architecture was partially effaced by sheets of epithelioid cells with indistinct borders, large nuclei and abundant eosinophilic cytoplasm (Figure 2A). The background showed small lymphocytes and numerous plasma cells. Immunohistochemistry revealed S100 expression in the majority of tumour cells (Figure 2B), granular cytoplasmic positivity for CD68 (Figure 2C), and weak, focal expression of CD45 (Figure 2D). Follicular dendritic cell markers (CD21 and CD23) and CD1a were negative, while additional markers were assessed to support the diagnosis and distinguish it from other dendritic cell neoplasms (Figure S1 and Table S1). The proliferative index (Ki67) was variable and ranged from 40% to 60%. Initial staging with computed tomography (CT) and fluorodeoxyglucose positron emission tomography (FDG-PET) revealed no additional sites of disease, and the patient was managed with active surveillance.

In April 2021, FDG-PET demonstrated a hypermetabolic large abdominal nodal conglomerate (SUVmax 31). Contrast-enhanced CT imaging confirmed widespread lymphadenopathy involving the right cervical region (30 mm), the area posterior to the left thyroid lobe (23 mm), the mediastinum (20 mm), and extensive intra-abdominal nodal disease extending from the celiac plexus to the pelvis (Figure S2A).

**Figure 3:**
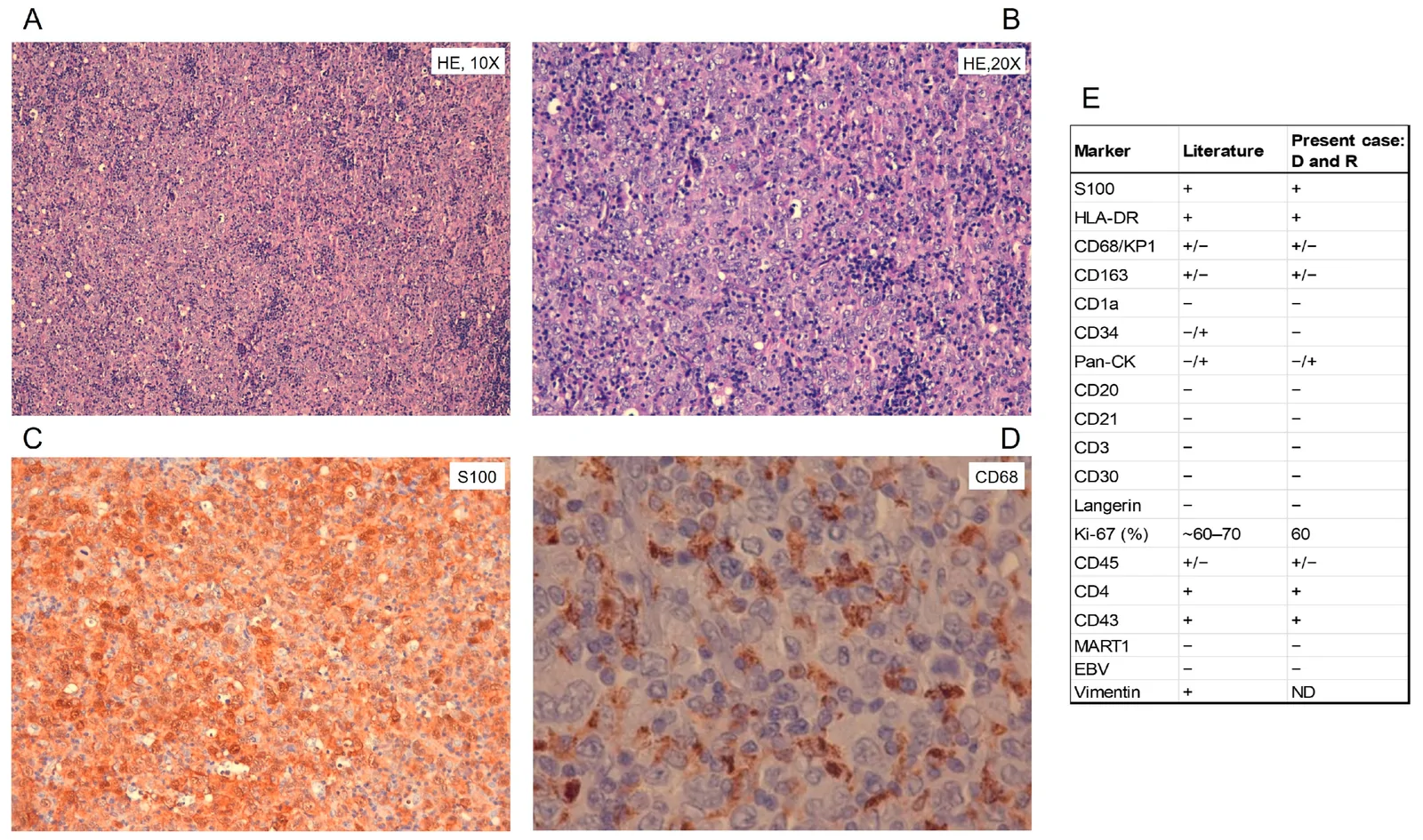
Abdominal mass lymph node sample at disease relapse. (**A**) Haematoxylin and eosin staining at 10× and (**B**) 20× magnification. Immunohistochemical analyses of (**C**) S100 (20×) and (**D**) CD68 (63×). (**E**) Summary of the immunohistochemical features of the patient’s samples at diagnosis and relapse compared with those reported in the literature. Given the comparable immunohistochemical profiles observed at the two time points, the findings are reported together (D: cervical lymph node biopsy obtained at diagnosis; R: lymph node biopsy obtained from an abdominal mass at relapse; ND: not determined).

An excisional biopsy of the abdominal mass performed in July 2021 confirmed relapsed IDCS, with morphologic and immunohistochemical features overlapping those observed at diagnosis (Figure 3A–E). Bone marrow biopsy showed no evidence of involvement.

At the time of recurrence, the patient was symptomatic, presenting with abdominal pain, weight loss of over 10 kg in one month, profuse night sweats, and severe asthenia. Routine laboratory tests revealed no significant abnormalities, with a normal complete blood count and serum lactate dehydrogenase levels within the reference range.

To identify potentially actionable molecular alterations and inform alternative therapeutic strategies in the event of refractory disease or relapse, comprehensive molecular profiling was performed on Formalin-Fixed, Paraffin-Embedded tissue from a meso-jejunal lymph node biopsy with an estimated tumour cell content of 100%. DNA sequencing was conducted using the TruSight Oncology (TSO) 500 panel (Illumina data sheet M-GL-00173 v4.0 and TSO500 Local App v.2.0.1.4), which enables the detection of single-nucleotide variants (SNVs), copy number alterations, microsatellite instability (MSI) status, and tumour mutational burden (TMB) within a single assay from Formalin-Fixed, Paraffin-Embedded tissue. Variant filtering excluded common polymorphisms (population frequency >1%) and synonymous variants, retaining splice-site or exonic variants with ≥100× coverage, quality score ≥20 and variant allele frequency >1.5% (annotated as “blacklist”). Variants meeting these criteria but flagged as “blacklist” were excluded, except for those also detected by RNA sequencing (n = 1; coverage 227 reads). RNA sequencing of 1385 cancer-related genes was carried out using the TruSight RNA Pan-Cancer Panel (Illumina). Fusion transcripts were identified using an in-house pipeline integrating four fusion-calling tools (STAR-Fusion v1.6.0 [12], FusionCatcher v1.20 [13], RNA-Seq Alignment v2.0.2 [14] and TopHat-Fusion v2.0.7 [15]) [16]. Variant calling from RNA-seq data was performed with ”RNA-Seq Alignment” v2.0.2 [14]. Variants from DNA (Table S2) and RNA (Table S3) datasets were annotated using Varsome (version 13.15.2), and clinical interpretation was primarily based on the AMP/ASCO/CAP guidelines for the interpretation and reporting of somatic variants [17]. Variants were retained if classified as AMP Tier I–III or not otherwise classified (NA) [17]. Copy number and gene fusion analyses did not reveal significant alterations. Microsatellite instability status was stable (3.45%), while tumour mutational burden (TMB) was high (21.3 mutations/Mb). Among genes covered by both panels (n = 384; Table S4), three variants were detected at both DNA and RNA levels (Tables S2 and S3). The most clinically relevant alteration was a canonical *KRAS* variant (c.35G > A; p.G12D). Additional variants involved *BCL10* and *SPEN* (Tables S2 and S3). DNA sequencing identified 19 variants (nine missense, seven frameshift and three nonsense), including four somatic alterations with strong clinical significance: two in *BRAF* (p.I342FfsTer30 and p.L597R), one in *KRAS* (p.G12D), and one in *SMARCA4* (p.R1189Q). The canonical *BRAF* V600 mutation was detected neither at diagnosis nor at relapse. From August to December 2021, the patient received six cycles of CHOP chemotherapy; after two cycles, an early disease reassessment showed a response to the ongoing treatment. At the end of the induction, the patient achieved complete metabolic and radiological remission (Figure S2B). There were no significant complications during induction therapy.

Given the aggressive disease presentation and the patient’s good performance status, consolidation with high-dose chemotherapy followed by ASCT was pursued. Stem cell mobilisation with IEV (ifosfamide, epirubicin, and etoposide) yielded 6.8 × 10^6^ CD34+ cells/kg without plerixafor. ASCT with BEAM conditioning (carmustine, etoposide, cytarabine, and melphalan) was performed in March 2022 and was well tolerated, without unexpected complications. Post-transplant FDG-PET confirmed complete remission. At the last follow-up, 54 months after ASCT, the patient remains in sustained complete remission.

## 3. Discussion

The present case illustrates two clinically relevant aspects of relapsed IDCS: the potential role of ASCT as consolidation after salvage chemotherapy and the value of comprehensive genomic profiling to expand the biological characterisation of this ultra-rare malignancy.

Although chemotherapy remains the most frequently adopted treatment for disseminated IDCS, no standard approach has been established for relapsed disease, and evidence supporting hematopoietic stem cell transplantation is limited to isolated reports [1,6]. Our patient presented with several adverse clinical features, including early relapse after surgical excision and bulky intra-abdominal disease, yet achieved a complete metabolic response following CHOP chemotherapy. Together with the excellent performance status, literature reports provided the rationale for consolidation with ASCT [6,8,9]. The durable complete remission maintained for 54 months suggests that high-dose chemotherapy followed by ASCT may represent an effective consolidation strategy in carefully selected patients with chemosensitive relapsed disease.

Beyond its clinical outcome, this case further expands the emerging molecular landscape of IDCS. Comprehensive genomic profiling identified concurrent alterations involving *KRAS*, *BRAF*, and *SMARCA4*, together with elevated TMB. While recurrent activation of the MAPK pathway has increasingly been recognised as a hallmark of histiocytic and dendritic cell neoplasms [7,8,9,10], the coexistence of a canonical *KRAS* G12D mutation and the non-canonical *BRAF* L597R variant has not, to our knowledge, been previously reported in IDCS [18]. Unlike the classical *BRAF* V600E mutation, L597R, also located in exon 15 within the catalytic domain of the serine/threonine kinase, belongs to class II BRAF alterations and has been associated with sensitivity to MEK inhibition in melanoma and other solid tumours, raising the possibility of targeted therapeutic strategies in the event of future disease recurrence [18,19,20]. Notably, some reports have recently described successful treatment with BRAF and MEK inhibitors, either as monotherapy or in combination with chemotherapy [4,21].

In addition, we identified a missense *SMARCA4* loss-of-function variant affecting the ATP-binding domain of this chromatin-remodelling protein [22,23]. Although its pathogenic significance in IDCS remains uncertain, SMARCA4 deficiency has been implicated in genomic instability and has been associated with therapeutic vulnerabilities in several malignancies, including Burkitt Lymphoma [20,23,24,25]. Particularly, *SMARCA4* inactivation has been associated with restored sensitivity to tyrosine kinase inhibitors in non-small cell lung cancer [26,27,28]. To our knowledge, this represents the first report describing a *SMARCA4* alteration in IDCS, further broadening the spectrum of genomic abnormalities reported in this disease.

Another intriguing finding was the elevated TMB. Although TMB is not routinely assessed in hematologic neoplasms, the few published IDCS cases evaluated by NGS have reported either low mutational burden or exceptionally ultra-hypermutated tumours [8,29]. Whether TMB has prognostic or predictive value in IDCS remains unknown. Nevertheless, given the growing evidence linking elevated TMB to improved responses to immune checkpoint inhibitors in solid tumours and the emerging activity of immunotherapy in malignant histiocytic neoplasms, this biomarker warrants further evaluation in future studies [29,30,31].

Interestingly, the prolonged remission observed after ASCT raises the hypothesis that durable disease control may not solely reflect the cytoreductive effect of high-dose chemotherapy. High-dose therapy induces immunogenic cell death and tumour antigen release, whereas immune reconstitution after ASCT promotes recovery and diversification of T cell immunity [32,33]. Whether these mechanisms may synergise with tumours harbouring increased neoantigen burden remains speculative but represents an attractive hypothesis deserving further investigation.

Overall, this case provides additional evidence supporting ASCT as consolidation for selected patients with relapsed chemosensitive IDCS while expanding the molecular spectrum of this ultra-rare neoplasm. More importantly, it underscores the importance of comprehensive genomic profiling in routine clinical practice, both to improve biological understanding and to identify potentially actionable alterations that may inform future precision medicine strategies.

## Data Availability

The original contributions presented in this study are included in this article/supplementary material. The raw sequencing data generated in this study are available through the European Genome-Phenome Archive (https://ega-archive.org/), under accession number EGAD50000003184.

## Supplementary Materials

The following supporting information can be downloaded at https://www.mdpi.com/article/10.3390/ijms27219474/s1.

## Abbreviations

The following abbreviations are used in this manuscript:

IDCS	interdigitating dendritic cell sarcoma
ASCT	autologous stem cell transplant
NGS	next-generation sequencing
CT	computed tomography
FDG-PET	fluorodeoxyglucose positron emission tomography
TMB	tumour mutational burden
TSO	TruSight Oncology
MHNs	malignant histiocytic neoplasms
