# Supplementary material for: Comprehensive Genomic Profiling of Relapsed Interdigitating Dendritic Cell Sarcoma Reveals Novel Molecular Insights in a Patient Achieving Durable Remission After Autologous Stem Cell Transplantation
Source: Int J Mol Sci. 2026 Oct 23;27(21):9474. doi: 10.3390/ijms27219474 (PMC13763408; doi:10.3390/ijms27219474)
Supplement: Supplementary file 1 [file ijms-27-09474-s001.zip › Supplementary figures and tables legends.pdf]

Supplementary Materials

Table S1. Immunohistochemical markers for the differential diagnosis of interdigitating dendritic cell sarcoma (IDCS). The table summarises the main immunohistochemical markers used to distinguish IDCS from other histiocytic and dendritic cell neoplasms and relevant differential diagnoses, along with the results obtained in the present case at the time of the initial diagnosis in a cervical lymph node.

**Differential diagnosis**	**HIC expectation**	**Case in exam**
melanoma	HMB45+	HMB45-
follicular dendritic cell sarcoma	CD21+ CD23+	CD21- CD23-
langheran cell sarcoma/histiocytosis	CD1a+	CD1a-
anaplastic large cell lymphoma	S100- CD30+	S100+ CD30-
carcinoma	CK+	CK-
histiocytic sarcoma	S100+/- CD163+	S100+ CD163-

Table S2. TruSight Oncology 500 (Illumina) single-nucleotide variant (SNV) detection in the FFPE tumour DNA sample. SNV annotation was performed using the TSO500 Local App (version 2.0.1.4) and Varsome (version 13.15.2). Variants annotated as "blacklist" were excluded unless confirmed at the RNA level (indicated in italics for genes shared between the two panels). Only variants classified as AMP Tier I–III or not assigned (NA) were retained. Variants also detected at the RNA level (in genes shared between the two panels) are indicated in italics.

Table S3. TruSight RNA Pan-Cancer Panel (Illumina) single nucleotide variant (SNV) detection in the FFPE tumour RNA sample. SNV annotation was performed using the RNA Sequencing Alignment application (BaseSpace, Illumina) and Varsome (version 13.15.2). Only variants classified as AMP Tier I–III or not assigned (NA) were retained. Variants also detected at the DNA level (in genes shared between the two panels) are indicated in italics.

Table S4. Comparison of genes included in the TruSight Oncology 500 (DNA) and TruSight RNA Pan-Cancer (RNA) panels (Illumina). 'x' indicates genes covered for the specified alteration type; '-' indicates no coverage; '*' indicates small variants found in gVCF file only (as indicated in the panel datasheet).

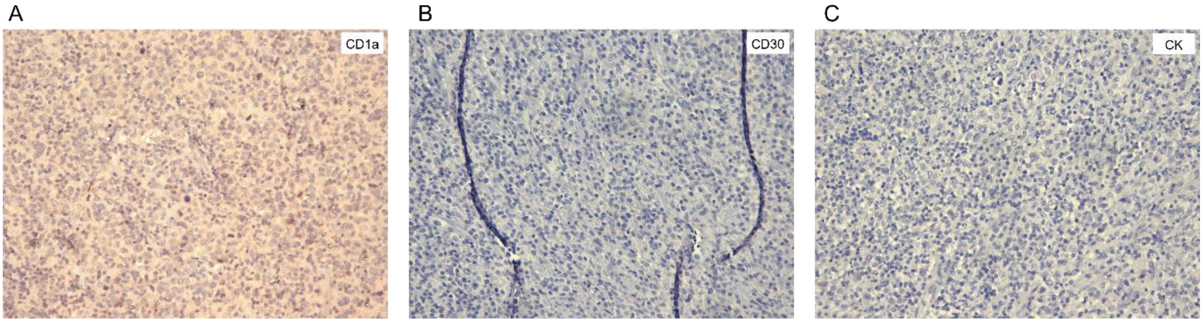

Figure S1. Additional negative immunohistochemical markers used for the differential diagnosis: results in the cervical lymph node sample at initial diagnosis. **A** CD1a (20x). **B** CD30 (20x). **C** CK(40x).

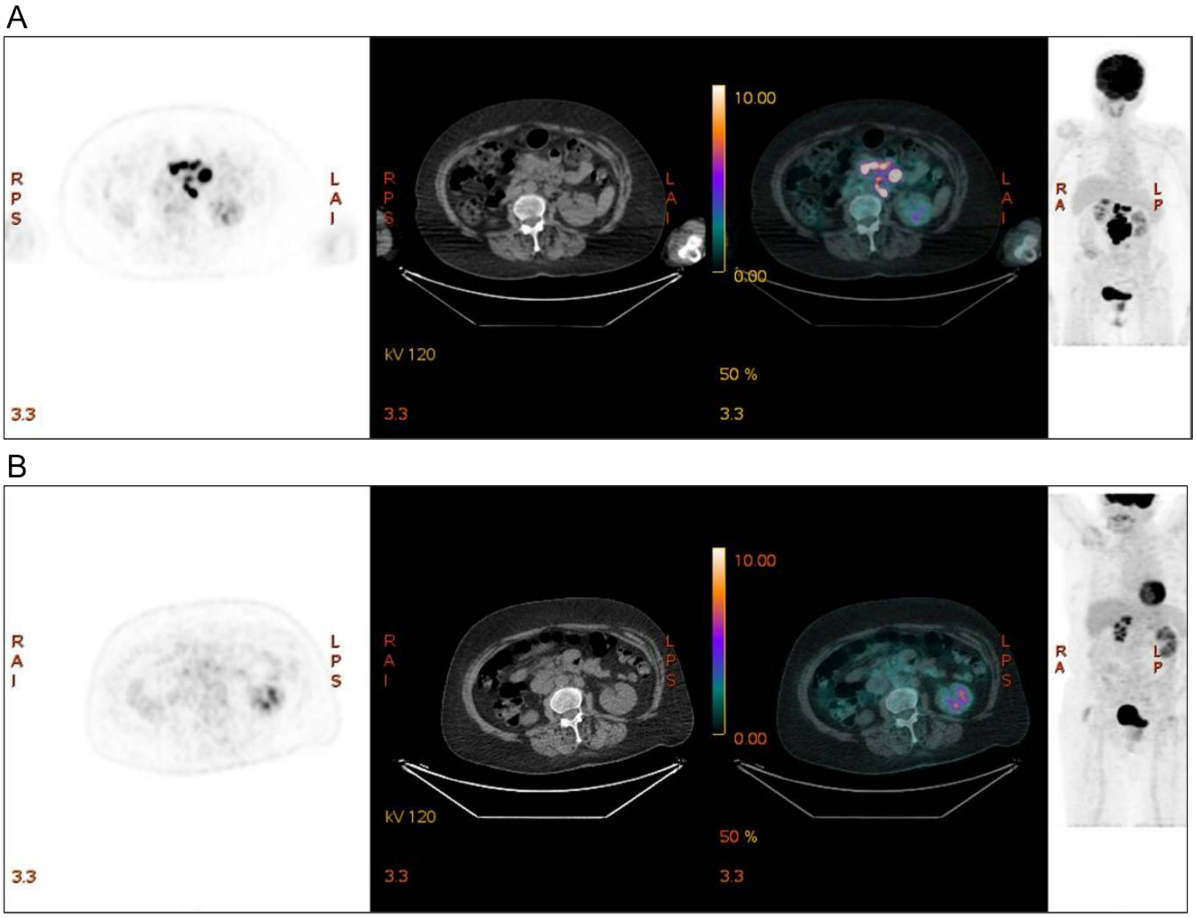

Figure S2. A FDG-PET pre-CHOP chemotherapy (April 2021) head-neck and thorax: No significant uptake (a metabolic activity below the significance threshold is present in the Barety space, with SUV max 2.65). Abdomen and pelvis: Hypermetabolic large nodal conglomerate in the left para-aortic and peripancreatic-mesenteric regions (SUV max 31.92). Skeletal system: No significant uptake. **B** FDG-PET post-CHOP chemotherapy (January 2022) Head-neck and Thorax: No significant uptake. Abdomen and pelvis: Complete regression of the hypermetabolic lymphadenopathy previously evident in the left para-aortic and peripancreatic-mesenteric regions. Skeletal system: No significant uptake. In conclusion, the PET scan is indicative of complete metabolic remission. FDG: Fluorodeoxyglucose. PET: Positron Emission Tomography. SUV: Standardised uptake value.
